# Second-Order Footsteps Illusions

**DOI:** 10.1177/2041669515622085

**Published:** 2015-12-24

**Authors:** Akiyoshi Kitaoka, Stuart Anstis

**Affiliations:** Department of Psychology, Ritsumeikan University, Kyoto, Japan; Department of Psychology, University of California, San Diego, CA, USA

**Keywords:** Motion, illusion, footsteps, second-order, abutting gratings, texture perception, reverse phi

## Abstract

In the “footsteps illusion”, light and dark squares travel at constant speed across black and white stripes. The squares appear to move faster and slower as their contrast against the stripes varies. We now demonstrate some second-order footsteps illusions, in which all edges are defined by colors or textures—even though luminance-based neural motion detectors are blind to such edges.

Anstis (2001, 2003, 2004) reported some illusory changes in apparent speed: In Movie #1, four squares, two of them light yellow and two of them dark blue, move horizontally at constant speed across stationary, vertical black and white stripes. Each square has the same width as two stripes, so that its front and back edges always lie on the same color (black or white). As Anstis found, the squares appear to speed up and slow down in alternation, depending upon their local contrast. When the dark blue squares lie on white stripes, they have high contrast (dark vs. white) and they appear to speed up momentarily. When they lie on black stripes, they have low contrast (dark vs. black) and they appear to slow down. The opposite is true for the light yellow squares.

Consequently, the squares appear to go faster and slower in alternation, like a pair of walking feet. So it was called the “footsteps illusion.” In Movie #1, the contrast hugely alters the apparent speed in real time. For instance, the two squares in each row seem to be alternately closer together and further apart, although their actual separation is always constant.

Anstis attributed the footsteps illusion to Thompson’s (1982) report that apparent speed varies with stimulus contrast. A low-contrast edge appears to move more slowly than a high-contrast edge. Howe, Thompson, Anstis, Sagreiya, and Livingstone (2006) emphasized other factors, including the contrast of the top and bottom edges of the moving squares. They also introduced variations, which they dubbed the belly dancer and Wenceslas illusions, plus a “kickback” illusion, which they attributed to reverse phi (Anstis, 1970; Anstis & Rogers, 1975; Rogers & Anstis, 1975).

Sunaga, Sato, Arikado, and Jomoto (2008) argued that motion detectors cannot play an important role in the footsteps illusion because the illusory misalignment between a light and a dark square was even more prominent for static than for moving squares. Also, squares that moved across a slowly flickering background did undergo contrast variations but showed little apparent speed variations. The authors regarded the footsteps illusion as a static geometrical illusion induced by the striped background, with motion detectors playing a minor role at best. These issues are still unresolved.

Meanwhile, we now report several second-order versions of the footsteps illusion, which are presented one after another, all within one large movie file. In Movie #1, the contrasts that determine apparent speed are defined by luminance (black, white, gray, etc.): moving yellow squares have high contrast against black stripes but low contrast against white stripes. The opposite is true for blue squares.

In the second-order Movies #2 to #5, the contrasts are defined by different visual properties, as shown in the thumbnail sketches the lower part of Figure 2. Thus, 

Movie #2 (contrast-modulated textures): The low-contrast moving squares have high modulation-contrast against the high-contrast textured stripes but low contrast against the low-contrast textured stripes.

Movie #3 (color): The magenta squares have low color-contrast against the red stripes but high color-contrast against the green stripes. The opposite is true for the cyan squares.

Movie #4 (fine vs. coarse): The finely textured moving squares have high spatial-frequency contrast against the fine stationary stripes but low contrast against the coarse stripes.

Movie #5 (discontinuous abutting gratings): The short horizontal lines in the moving squares have large (small) offset-contrast when they are strongly misaligned (almost aligned) with horizontals in the stationary stripes. Figure 1 illustrates discontinuous abutting gratings (Kanizsa, 1974; Soriano, Spillmann, & Bach, 1996; von der Heydt & Peterhans, 1989). The greater the offset, the higher the virtual “contrast” of the edges.

**Figure 1.**
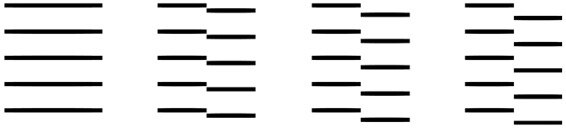
Second-order vertical edges defined by discontinuous abutting gratings. Their virtual contrasts are (a) zero, (b) low, (c) medium, and (d) high.

Figure 2 shows observers’ ratings of the strength of these second-order illusions.

**Figure 2.**
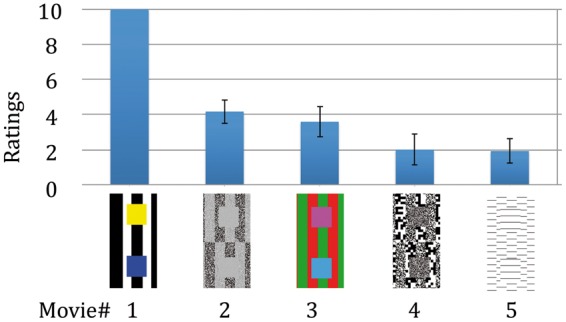
Rated strengths of the second-order footsteps illusions in Movies #2 to #5 when movies were viewed at a distance of 1.5 m and the footstep effect of Movie #1 was rated “10.”

In all these movies, the squares always move from side to side at constant speed, but they seem to vary their apparent speeds and separations as they move across the stripes. Figure 2 shows ratings of the strength of the various second-order footsteps (mean + *SE* for 12 observers), with the footstep effect of Movie #1 being rated “10.” The illusions are weaker but nonetheless present. Yet, the motion detectors first reported by Reichardt (1961) and modeled by Adelson and Bergen (1985) are blind to the motions of most of the edges in Movies #2 to #5. Thompson (1982) showed that the apparent speed of first-order motion is dependent upon contrast. Our results suggest that the same is true for second-order motion, which may impose constraints upon models of second-order motion perception.

## Supplementary Material

Supplementary material
